# Relationship between blood pressure and cognitive decline: A 4‐year community‐based prospective cohort study

**DOI:** 10.1002/alz70858_098728

**Published:** 2025-12-25

**Authors:** Yanyu Wang, Yulu Yan, Suhang Shang, Ling Gao, Liangjun Dang, Shan Wei, Chen Chen, Jingyi Wang, Qiumin Qu, Yan Qu

**Affiliations:** ^1^ The First Affiliated Hospital of Xi'an Jiao Tong University, Xi'an, Shaanxi, China; ^2^ The First Affiliated Hospital of Xi’an Jiaotong University, Xi'an, Shaanxi, China; ^3^ The First Affiliated Hospital of Xi'an Jiaotong University, Xi'an, Shaanxi, China; ^4^ Huxian Hospital of Traditional Chinese Medicine, Xi'an, Shaanxi, China

## Abstract

**Background:**

To explore the relationship between baseline blood pressure and cognitive decline over 4 years in middle‐aged and elderly people in rural Xi'an.

**Method:**

This was a community‐based prospective cohort study. The population was selected using clustering sampling method and people who lived in the Qubao village of Xi’an aged 40 years or older were all enrolled. The people with normal cognition was surveyed between October 2014 and March 2015, and two follow‐up visits were conducted in 2016 and 2018. The Mini‐Mental State Examination (MMSE) was used to assess the cognition, and a drop of ≥4 points in MMSE score was defined as significant cognitive decline in 4‐years follow‐up. Univariate analysis and multivariate binary Logistic regression were used to analyze the relationship between blood pressure and cognitive decline.

**Result:**

A total of 1350 subjects were included in the analysis, including 235 subjects (17.4%) with baseline age ≥65 years, 533 subjects (39.5%) male, 671 subjects (49.7%) with hypertension, and 840 subjects (62.2%) with high systolic blood pressure(SBP), and 395(29.3%) had high diastolic blood pressure(DBP). During the 4‐year follow‐up, 56 cases (4.2%) met the criteria of cognitive decline. No significant association was found between hypertension, or high SBP and cognitive decline in the general population, < 65‐year‐old subgroup and ≥65‐year‐old subgroup. The incidence of cognitive decline was higher in high DBP group than that in the normal DBP group (5.1% vs. 2.7%, *p* = 0.043). Multivariate analysis showed that high DBP was not associated with cognitive decline in the general population (OR = 1.744, 95% CI: 0.953‐3.192, *p* = 0.071) and in the subgroup aged ≥65 years (OR = 0.858, 95% CI: 0.221‐3.338, *p* = 0.825), but was associated with cognitive decline in < 65‐year‐old subgroup (OR = 2.051, 95% CI: 1.005‐4.186, *p* = 0.048).

**Conclusion:**

High DBP was associated with a cognitive decline in 4‐year in people aged 40‐65 years, but not in those aged ≥65 years. These indicated that high DBP may be a risk factor for cognitive decline in middle‐age people.

